# A protein–protein interaction underlies the molecular basis for substrate recognition by an adenosine-to-inosine RNA-editing enzyme

**DOI:** 10.1093/nar/gky800

**Published:** 2018-09-07

**Authors:** Suba Rajendren, Aidan C Manning, Haider Al-Awadi, Kentaro Yamada, Yuichiro Takagi, Heather A Hundley

**Affiliations:** 1 Department of Biology, Indiana University, Bloomington, IN 47405, USA; 2 Medical Sciences Program, Indiana University, Bloomington, IN 47405, USA; 3 Department of Biochemistry and Molecular Biology, Indiana University School of Medicine, Indianapolis, IN 46202, USA

## Abstract

Adenosine deaminases that act on RNA (ADARs) convert adenosine to inosine within double-stranded regions of RNA, resulting in increased transcriptomic diversity, as well as protection of cellular double-stranded RNA (dsRNA) from silencing and improper immune activation. The presence of dsRNA-binding domains (dsRBDs) in all ADARs suggests these domains are important for substrate recognition; however, the role of dsRBDs *in vivo* remains largely unknown. Herein, our studies indicate the *Caenorhabditis elegans* ADAR enzyme, ADR-2, has low affinity for dsRNA, but interacts with ADR-1, an editing-deficient member of the ADAR family, which has a 100-fold higher affinity for dsRNA. ADR-1 uses one dsRBD to physically interact with ADR-2 and a second dsRBD to bind to dsRNAs, thereby tethering ADR-2 to substrates. ADR-2 interacts with >1200 transcripts *in vivo*, and ADR-1 is required for 80% of these interactions. Our results identify a novel mode of substrate recognition for ADAR enzymes and indicate that protein–protein interactions can guide substrate recognition for RNA editors.

## INTRODUCTION

Diverse enzymes catalyze modification of nucleotides in all types of nucleic acids present in the cell. While transfer RNAs (tRNAs) are the most extensively modified molecules in the cell (1), recent technological developments have enabled the detection of several types of modifications in eukaryotic messenger RNAs (mRNAs) (2,3). Adenosine deamination is the most frequent RNA modification that directly alters genetic information and results in functional consequences on gene expression (4). Deamination of adenosine (A) results in inosine (I), which has similar base-pairing properties as guanosine. Due to these differences in base pairing, A-to-I editing can alter the amino acid encoded by a codon, modify splice sites and affect the interaction of the RNA molecule with itself or other RNAs, such as microRNAs (5). In addition, A-to-I editing in long double-stranded regions has been reported to prevent silencing of host RNAs and improper activation of the immune response by self-RNAs (6–8).

Loss of the enzymes responsible for A-to-I editing, adenosine deaminases that act on RNA (ADARs), results in lethality in mice and behavioral phenotypes in worm and fly model systems (9–13). Consistent with important roles in normal development and proper neuronal function, alterations in RNA editing occur in over 35 human pathologies, including several neurological disorders, metabolic diseases and cancer (14–17). While much effort has focused on identifying the altered editing that occurs in disease, the molecular mechanisms that alter ADAR substrate recognition and RNA editing efficiency in disease are largely unknown (18). As spatiotemporal editing patterns vary for individual genes and do not directly correlate with levels of the transcript or editing enzymes (19,20), specific factors and/or distinct mechanisms of substrate recognition may be important for regulating ADAR editing *in vivo*.

Substrate recognition by ADARs requires the ability to both bind specific mRNAs and select target adenosines within the double-stranded region for deamination (21). Biochemical and structural studies indicate that the deaminase domain interacts with double-stranded RNA (dsRNA) and contributes to the selection of specific adenosines to edit (22–24). However, chimeric studies between ADARs and other dsRNA-binding proteins (dsRBPs) indicate that dsRBDs contribute to efficient editing *in vitro* (25), and biochemical studies of ADARs show the dsRBDs are important for providing both high-affinity binding and selectivity (26–28). While these data suggest both the dsRBDs and the deaminase domains regulate aspects of ADAR substrate recognition *in vitro*, there is very little information on how ADAR enzymes select some targets and discriminate against others *in vivo*. Early studies of *Xenopus* ADAR1 demonstrated that the deaminase domain was dispensable for ADAR1 binding to nascent transcripts *in vivo*, whereas individual deletions of each of the three dsRBDs resulted in differential transcript localization (29). While these data suggest the dsRBDs present in ADARs may contribute to *in vivo* substrate specificity, dsRBDs can also mediate protein–protein interactions between ADARs (30,31) and other dsRBPs (31–35). Therefore, consequences of loss of dsRBDs on *in vivo* dsRNA binding by ADARs may be caused by loss of specific target recognition by the dsRBD or loss of interaction with important, but yet unidentified co-factors.

To gain insight into the molecular mechanisms of ADAR substrate recognition, we compared *in vitro* and *in vivo* recognition of dsRNA. We focused on the *Caenorhabditis elegans* ADARs as worms lacking A-to-I editing are viable (12), thus providing the opportunity to assess wild-type (WT) and mutant ADAR proteins *in vivo*. The *C. elegans* genome encodes two proteins, ADR-1 and ADR-2, with the common ADAR family domain structure, but previous studies have shown that ADR-2 is the only active A-to-I editing enzyme in worms (12,36). Although ADR-1 is a deaminase-deficient ADAR family member, ADR-1 promotes editing by ADR-2 at specific adenosines across the transcriptome (36). The ability of ADR-1 to promote editing by ADR-2 is critical for proper neuronal function, as evidenced by our recent finding that both ADR-1 and ADR-2 are required for proper editing and neural expression of an mRNA that is needed for proper chemotaxis of worms (37). To understand the mechanistic function of these two proteins in A-to-I editing, we developed an expression system that enables production of *C. elegans* ADAR proteins in recombinant forms. By analyzing WT and mutant ADAR proteins *in vitro* as well as *in vivo*, we demonstrate that ADR-1 and ADR-2 have a direct protein–protein interaction that involves the second dsRBD of ADR-1. In addition, using an RNA immunoprecipitation (RIP) assay for ADR-1 and biochemical editing assays, we determined that ADR-1 primarily binds mRNA through its first dsRBD, and binds ADR-2 with its second dsRBD, and both ADR-1 dsRBDs are required to promote editing by ADR-2. ADR-2 RIP coupled to high-throughput sequencing indicated that ADR-1 was required for ADR-2 to stably associate with 983 of 1235 transcripts in WT worms. Together, these findings indicate that ADR-1 acts as an endogenous co-factor to direct ADR-2 to specific substrates *in vivo*, and opens the possibility that ADAR-editing enzymes in humans could interact with other RNA-binding proteins, including deaminase-deficient ADAR family members, to recognize different target mRNAs *in vivo*.

## MATERIALS AND METHODS

### Strains and culture

Worms were maintained under standard laboratory conditions on Nematode growth media (NGM) plates seeded with *Escherichia coli* OP50 (38). Transgenic worm lines were generated by microinjection into the gonads of young adult worms of the appropriate genetic background as described previously (39). The injection contained the following: 1 ng/μl of the *adr-1* transgene of interest, 20 ng/μl of the dominant marker *rab3::gfp::unc-54 (3*′ *UTR)* and 79 ng/μl of 1 kb DNA ladder (NEB). Transgenic strains were maintained by passaging worms expressing the Green Fluorescent Protein (GFP) marker. As described previously (39), the transgenes expressing *adr-1* were injected with a modified pBluescript SK plasmid that contained the genomic *adr-1* locus, including the *adr-1* promoter (1245 nt upstream of the start codon) and 3′ UTR (1560 nt downstream of the stop codon) and three copies of the FLAG epitope (DYKDHD) immediately after the start codon. Mutations to the dsRBDs of *adr-1* were generated by polymerase chain reaction (PCR) and confirmed by Sanger sequencing. The following transgenic worm strains were utilized in this study and previously published: BB21 *adr-1(tm668)* + *blmEx1[3XFLAG-adr-1* genomic, *rab3::gfp::unc-54 (3*′ *UTR)]* (39) and BB21 *adr-1(tm668)* + *blmEx2[3X FLAG-adr-1* genomic with*dsRBD1 (K223E, K224A and K227A)* and*dsRBD2 (K583E, K584A and K587A), rab3::gfp::unc-54 (3*′ *UTR)*] (36). Newly generated transgenic lines include BB21 *adr-1(tm668)* + *blmEx11*[*3XFLAG-adr-1* genomic with mutations in *dsRBD1 (K223E, K224A, and K227A*), *rab3::gfp::unc-54 (3*′ *UTR)*] and BB21 *adr-1(tm668)* + *blmEx12*[*3X FLAG-adr-1* genomic with mutations in*dsRBD2 (K583E, K584A and K587A), rab3::gfp::unc-54 (3*′ *UTR)*].

Sf9 and Hi5 insect cell cultures were maintained as described in the Invitrogen instruction manual, ‘Guide to Baculovirus Expression Vector Systems (BEVS) and Insect Cell Culture Techniques’ and (40), respectively.

### RNA immunoprecipitation (RIP) assay

RIP assays for ADR-1 and ADR-2 were performed as described previously (36,37). Random hexamers (Thermo-Scientific) or gene-specific primers (listed in Supplementary Table S5) were used to synthesize complementary DNA (cDNA) for the FLAG-ADR-1 RIP and ADR-2 RIP assays, respectively. The RNA used for quantitative real-time PCR (qPCR) was DNase-treated and stored at −80°C. The qPCR reactions used gene-specific primers (see Supplementary Table S5 for sequence information, amplicon length and location) was performed to quantify *lam-2* (WormBase ID: WBGene00016913), *pop-1* (WormBase ID: WBGene00004077), *C35E7.6* (WormBase ID: WBGene00016458) and *clec-41* (WormBase ID: WBGene00007153) cDNA abundance in both total RNA (input) and immunoprecipitated RNA from the indicated strains. qPCR reactions were performed on 1/10 of the total reverse transcription volume for each sample, with two technical replicates for each qPCR reaction. The total volume of the qPCR reaction was 10 μl, used the SybrFast Master Mix (Kapa Biosystems, cat no: KK4602) and was performed on an Eppendorf Realplex Mastercycler using the PCR program (step 1: 95°C for 3 min, steps 2–4: 95°C for 10 s, 61°C for 15 s, 72°C for 20 s, repeat steps 2–4 for 40 cycles, step 5: melting curve). All qPCR quantification for each biological replicate included eight wells of the amplicon of interest (standards), which were 10-fold serial diluted and used to generate a standard curve of cycle threshold versus the relative concentration of amplicon. The standard curves were plotted with a logarithmic scale in regard to concentration and fit with a linear line. The fit of the lines (*R*^2^) ranged from 0.98 to 1.00, and all data points fell within the standard curve. All amplicons spanned an exon–exon junction (see Supplementary Table S5), which would give rise to an ∼150 bp PCR product from cDNA and a larger product if genomic DNA contamination was present in the RNA samples. Each qPCR reaction was examined for the presence of a singular peak in the melting curve step.

### ADR-2 RIP-Seq

RNA was isolated from input lysates and immunoprecipitates from WT, *adr-2(-)* and *adr-1(-)* worms as described above. Isolated RNA was subjected to ribosomal RNA depletion using Ribo-Zero gold kits (Human/Mouse/Rat: Illumina Ref: MRZ116C) according to the manufacturer’s instructions (except 1/8 of the recommended Ribo-Zero removal solution was used for IP samples due to the low amount of RNA present). The rRNA-depleted samples were used as the starting material to generate sequencing libraries using the KAPA stranded RNA-seq library preparation Kit (Illumina Ref: KK8400). Briefly, RNA samples were fragmented into 200–300 bp strands by high temperature (94°C for 6 min) and used to make first and second strands of cDNA. Adapters (KAPA S1 adapter kit Ref: 08005770001) were ligated to the cDNA and the libraries were amplified using minimal PCR cycles. Libraries were sequenced on an Illumina NextSeq500 instrument at the Indiana University Center for Genomics and Bioinformatics. Approximately 30–50 million 75 bp single-end sequence reads were obtained for each library.

### Bioinformatic analysis of RIP reads

In brief, 75 bp single-end, reversely stranded, RNA-Seq reads were trimmed of adapters and aligned to ce11(WS262) using the following STAR (v2.5.2b) parameters: *[outFilterMultimapNmax 1, outFilterScoreMinOverLread 0.66, outFilterMatchNminOverLread 0.66, outFilterMismatchNmax 10, outFilterMismatchNoverLmax 0.3]*. FeatureCounts (v1.5.2) was used to count mapped reads to Wormbase (WS262) gene annotations using the [-s 2] flag for reversely stranded reads. Genes with read counts of zero across all samples were removed as indicating non-expressed transcripts. To determine enrichment of transcripts in the IP, RNA-sequencing datasets for two biological replicates of both immunoprecipitate and input samples for each strain were analyzed. Raw read counts were input into DESeq2 (v1.18.1) to test for ratio of ratios, in this case ((IPA/InputA)/(IPB/InputB)), using a likelihood ratio test. To determine high confidence editing sites in the input RNA-Seq datasets from WT, *adr-2(-)* and *adr-1(-)*, the aligned reads for the two replicates of each sample were merged to increase coverage at edited sites. These sites were then identified using *SAILOR* as previously described (37). Sites with a confidence of ≥0.99 were called as high confidence for downstream analysis. Annotation of these sites was carried out with a custom python script using the wormbase WS262 annotations. Transcripts that had a significant difference in DESeq2 values (*P*_adj_ < 0.05, using Benjamini–Hochberg correction) between experimental (WT or *adr-1(-)*) and control (*adr-2(-)*) were considered to be enriched in the ADR-2 IPs. A list of *C. elegans* edited transcripts from several published RNA-seq datasets (37) was overlapped with the enriched transcripts to determine the proportion of mRNAs that have been previously identified as edited.

### Construction of baculovirus vectors for expression of ADR-1 and ADR-2

The coding region of *adr-1* (WormBase ID: WP: CE32459) was PCR amplified and cloned into the pFastBacHTB vector downstream of a 6-histidine tag and a TEV protease recognition site. Mutations to the individual dsRBDs of ADR-1 were generated using site-directed mutagenesis (KKxxK into EAxxA) and the double dsRBD mutant was generated by subcloning the dsRBD2 mutations into the dsRBD1 mutant plasmid. The coding region of *adr-2* (WormBase ID: WP: CE25120) was PCR amplified and cloned into the pLK2-10His-3C vector (41) immediately downstream of a 10-histidine tagged maltose-binding protein (MBP) and a human rhinovirus 3C protease site. Mutations to the dsRBD (KKxxK into EAxxA) of ADR-2 were generated using site-directed mutagenesis.

### Virus production and protein expression

Recombinant baculoviruses expressing ADR proteins were generated using methods described in the Invitrogen instruction manual, ‘Guide to Baculovirus Expression Vector Systems (BEVS) and Insect Cell Culture Techniques’ as well as the methods described in (40), respectively. Optimization of ADR-2 expression was carried out using Titer estimation for quality control (TEQC) method as described in (42).

### Protein purification and quantification

WT ADR-1 and ADR-1 dsRBD mutants were expressed in Sf9 cells. Baculovirus-infected cells were collected by centrifugation 72 h after infection. Cells were resuspended in Lysis Buffer (20 mM Tris–HCl [pH 8.0], 500 mM NaCl, 5 mM imidazole, 10% glycerol, 1% Triton-X-100) and sonicated for 20 s at an amplitude 20 for three times, resting on ice for 1 min between each sonication (Misonix-ultrasonic liquid processor). Following ultracentrifugation at 179,200 g for 30 min at 4°C, the supernatant was added to Ni-NTA (Qiagen) resin and incubated for 1 h. Resin was washed with buffer (20 mM Tris–HCl [pH 8.0], 500 mM NaCl, 35 mM imidazole, 10% glycerol, 1% Triton-X-100) and His-ADR-1 was eluted with buffer (20 mM Tris–HCl [pH 8.0], 500 mM NaCl, 500 mM imidazole, 10% glycerol, 1% Triton-X-100).

WT ADR-2 and ADR-2 mutants were expressed in Hi5 cells. Baculovirus-infected cells were collected by centrifugation 72 h after infection. Cells were resuspended in Amylose wash buffer (20 mM Tris–HCl [pH 8.0], 500 mM NaCl, 10% glycerol, 0.1% Triton-X-100), sonicated and centrifuged as described for recombinant ADR-1. The supernatant was incubated with Amylose resin (NEB) for 1 h and then washed with 5–10 column volumes of wash buffer. ADR-2 was cleaved from the amylose-bound MBP tag by 3C protease. Cleaved ADR-2 was diluted and then incubated with Heparin resin (GE Healthcare) and eluted using a high-salt buffer (20 mM Tris–HCl [pH 8.0], 500 mM NaCl, 10% glycerol, 0.5 mM 2-Mercaptoethanol).

Both ADR-1 and ADR-2 were dialyzed into storage buffer (20 mM Tris–HCl [pH 8.0], 200 mM KCl, 10% glycerol, 0.5 mM 2-Mercaptoethanol). Protein purity and concentration were determined by sodium dodecylsulphate-polyacrylamide gel electrophoresis (SDS-PAGE) and Coomassie staining, alongside known concentrations of bovine serum albumin (BSA-Sigma) standards. Proteins were concentrated using Amicon Ultra-4 centrifugal filters (30 K), aliquoted and stored at −80°C.

### Preparation of radioactively labeled dsRNA

The 200 bp long dsRNA was prepared by amplifying regions of the *C. elegans lam-2* 3′ UTR that contain 25 sites that undergo A-to-I editing *in vivo*, 8 of which require ADR-1 for maximal editing (36). PCR templates corresponding to the 5′ and 3′ half of the double-stranded 3′ UTR were generated with the forward primer for each template containing a T7 RNA polymerase binding site. PCR templates were individually transcribed in the presence of T7 RNA polymerase, Nucleoside triphosphate (NTPs) (100 mM CTP, GTP, UTP each and 5 mM ATP), and radioactive ^32^P α-ATP. After 2 h, a Chroma-spin column (Clontech) was used to remove unincorporated nucleotides. RNA concentration was calculated based on the radioactive ATP incorporated in each strand (measured by a scintillation counter). Equal molar ratios of each strand were combined and incubated in 1× annealing buffer (from 5× annealing buffer: 50 mM Tris–HCl [pH 7.5], 100 mM NaCl, 5 mM ethylenediaminetetraacetic acid (EDTA)) and then subjected to high heat (90°C) for 2 min in a water bath and cooled gradually to room temperature. The annealed dsRNA was subjected to Native PAGE at a constant power of 3 W for ∼3 h. The dsRNA band was excised from the Native gel, and the RNA was allowed to passively diffuse into 350 μl of 1× electrophoretic mobility shift assay (EMSA) buffer overnight. The concentration of the dsRNA was measured using a Beckman scintillation counter model LS600SC.

The 46 bp long dsRNA was made by annealing two complementary synthetic ssRNAs from *lam-2* 3′-UTR region (Supplementary Table S5). These ssRNAs were end labeled with ^32^P γ-ATP using Polynucleotide kinase (Polynucleotide kinase-NEB). Free nucleotides were removed using microspin columns (Biorad). Annealing of ssRNAs, gel extraction of dsRNA and quantification were performed as described above.

### Gel mobility shift assay

Gel mobility shift assays were performed as previously described (26). Briefly, 20 pM dsRNA was incubated with recombinant proteins in EMSA buffer (50 mM KCl, 10 mM Tris–HCl [pH 8.0], 10% glycerol, 1 mM MgCl_2_, 0.5 mM dithiothreitol [DTT]) on ice for 30 min. Reactions were stopped by adding 5× loading buffer (2.5× TBE, 50% glycerol) and 75% of the reaction was loaded into a Native 6% (29:1 acrylamide/bisacrylamide (Biorad)) gel. Electrophoresis was performed at 4°C at 200 V for 2–2.5 h with 0.5× Tris-borate-EDTA (TBE) running buffer. Gels were dried, exposed to phosphorimager screens overnight and autoradiographed (Typhoon FLA 9500). ImageQuant TL (1D v8.1) was used to quantify the bound and free RNA. RNA-binding isotherms were obtained using GraphPad Prism 7.0a with a curve fit of specific binding with Hill slope.

### *In vitro* deamination assay

Worm extracts were made as previously described (12). Briefly, worms were collected from NGM plates using 1× M9 buffer (0.04 M Na_2_HPO_4_, 0.02 M KH_2_PO_4_, 0.009 M NH_4_Cl, 0.02 M NaCl) and two volumes of TGKED buffer (50 mM Tris–HCl [pH 8.0], 50 mM KCl, 150 mM NaCl, 25% glycerol, 0.1 mM EDTA, 0.5 mM DTT) containing Complete protease inhibitor (Roche) were added to the worm pellet. The worm suspension was sonicated at an amplitude of 20 (power ∼4) for 10 s four times and at an amplitude of 35 four times (Misonix-ultrasonic liquid processor), resting the tube on ice for 1 min between each sonication. Lysates were centrifuged at 4°C at 16 000 g for 45 min and quantified using a Bradford assay. Extracts were aliquoted, flash frozen and stored at −80°C.

Deamination reactions (100 μl) were performed in assay buffer (40 mM Tris–HCl [pH 7.9], 5 mM EDTA, 25 mM KCl, 10 mM NaCl, 1.1 mM MgCl_2_, 5% glycerol, 1 mM DTT and 0.4 U/μl RNasin (Promega)) with a final concentration of 1 nM radiolabeled *lam-2* 3′ UTR (long) and 100–150 μg worm extracts. Reactions were incubated at 20°C for 2 h and stopped by the addition of a 10:1 Trizol:chloroform mixture. RNA was isolated from the aqueous layer, ethanol precipitated and subjected to cleavage with P1 nuclease (US Biological Life Sciences) at 37°C for 30 min. Cleaved nucleotides were extracted using a 25:24:1 phenol/chloroform/isoamyl mixture and then lyophilized using a speed vacuum. Mononucleotides were resuspended in water and spotted on a thin-layer chromatography (TLC) plate (PEI-cellulose, Selecto Scientific) that was run with solvent (197.5 ml saturated (NH_4_)_2_SO_4_, 47.5 ml 0.1 M NaOAc, pH 6.0, 5 ml isopropanol) for 2 h, exposed to phosphorimager overnight and autoradiographed (Typhoon FLA 9500).

### Co-IP and pulldown assays

For the *in vivo* co-IP assay, immunoprecipitates of ADR-1 with α-FLAG magnetic beads were performed as described above (RIP assay), except worms were not subjected to ultraviolet (UV) crosslinking. The immunoprecipitates were washed with RIP wash buffer, resuspended in 2× SDS buffer and subjected to SDS-PAGE. For the *in vitro* pulldown assay, Dynabeads (Invitrogen, anti-Rabbit IgG) were incubated with ADR-2 antibody (PA6496, Fisher Scientific). RIP wash buffer with freshly added detergents (0.1% NP-40, 0.5% Triton-X-100) was used to wash the antibody-bound beads before the addition of ADR-2 to the final concentration of 5 nM. After 2 h of incubation at 4°C, unbound ADR-2 was removed with wash buffer. BSA was added to the beads at a final concentration of 0.4 μg/μl to reduce non-specific binding and incubated for 1 h. The beads were then incubated with ADR-1 proteins (final concentration of 3 nM) in EMSA buffer (50 mM KCl, 10 mM Tris–HCl [pH 8.0], 10% glycerol, 1 mM MgCl_2_) for 1 h with or without 46 bp dsRNA (0–10 nM final concentration). The beads were washed with RIP wash buffer, resuspended in 30 μl of 2× SDS buffer and subjected to SDS-PAGE. Immunoblotting analysis for the *in vivo* IP utilized FLAG and ADR-2 (PA6496) antibodies, while the *in vitro* pulldowns utilized ADR-2 antibody (IU529) and a custom ADR-1 antibody previously described (39).

## RESULTS

### ADR-2, the only A-to-I editing enzyme in *C. elegans*, has a reduced affinity for substrate RNAs compared to other ADARs

**Figure 1.**
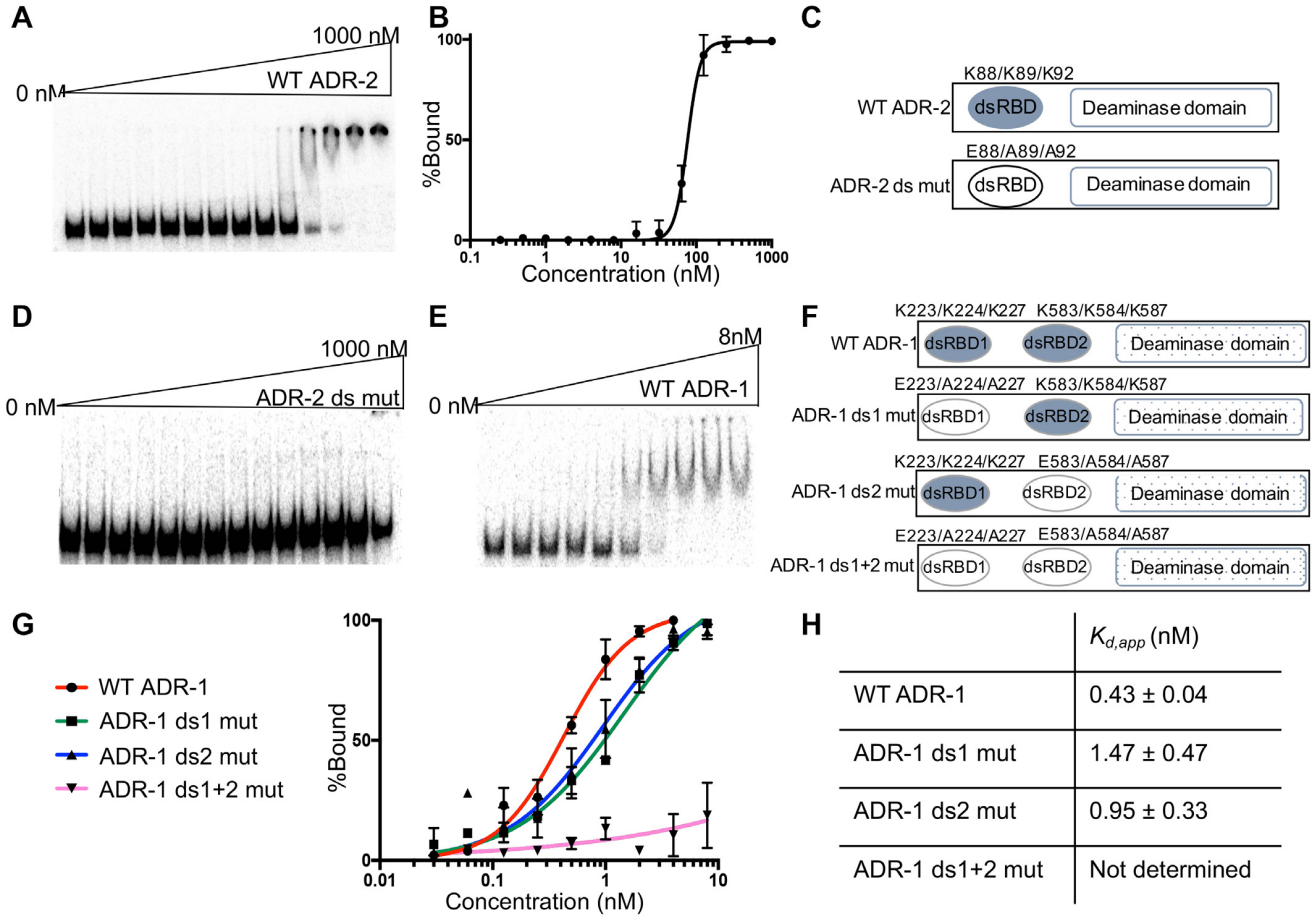
ADR-2 exhibits over a 100-fold weaker affinity to dsRNAs than ADR-1. (**A**) Increasing amounts of WT ADR-2 were mixed with ^32^P-labeled 200 bp dsRNA from the *lam-2* 3′UTR (20 pM) and incubated on ice for 30 min. Complex formation was analyzed by native gel electrophoresis. Initial and final protein concentrations are marked above gel with adjacent lanes representing a 2-fold difference in protein concentration. (**B**) Autoradiograms of the dried gels were used to determine the binding affinity of ADR-2 to dsRNA. The average value for % bound at each concentration of protein was used to generate the binding curve. Error bars indicate standard error mean (SEM) for each point from three replicates of the gel shift. The dissociation constant (*K*_d, app_) was calculated from the binding curve generated for specific binding using a Hill slope. (**C**) Schematic of the protein structure for WT ADR-2 and the ADR-2 dsRBD mutant (ds mut). The dsRBD is shown as an oval (WT in light blue and mutant in white) and the deaminase domain as a rectangle. The amino acid residues for the lysines within the conserved KKxxK motif are listed above the dsRBD as well as the specific mutations present in the dsRBD mutant. (**D**) A representative gel shift assay for the ADR-2 ds mut incubated with 20 pM of the 200 bp *lam-2* 3′ UTR dsRNA. (**E**) Increasing concentrations of WT ADR-1 were mixed with ^32^P-labeled 200 bp dsRNA corresponding to the sequence from the *lam-2* 3′-UTR (20 pM). Initial and final protein concentrations are marked above gel with adjacent lanes representing a 2-fold difference in protein concentration. (**F**) Schematic of the WT ADR-1 and ADR-1 dsRBD mutants. (**G**) RNA-binding isotherms for gel shift assays conducted with the 200 bp RNA and the indicated ADR-1 proteins. The average value for % bound at each protein concentration was used to generate the binding curve. Error bars indicate SEM for each point from three replicates of each assay, except ADR-1 ds1+2 mutant where *n* = 2. (**H**) Values for the dissociation constant (*K*_d,app_) were calculated from the RNA-binding isotherms shown in (G).

Deamination of dsRNA by ADARs requires binding of the editing enzyme to the substrate and base flipping of the target adenosine (43). To interrogate the biochemical properties of the *C. elegans* A-to-I editing enzyme, ADR-2, we established the first expression and purification protocol for *C. elegans* ADR-2 using a BEVS (Supplementary Figure S1A). Binding of recombinant ADR-2 to dsRNA was examined using a gel mobility shift assay with a 200 bp dsRNA corresponding to sequence from the 3′ UTR of *lam-2*, a known ADR-2 substrate (44). Increasing concentrations of ADR-2 (up to 1 μM) were incubated with the 200 bp dsRNA and the measured affinity (*K*_d_,_app_) was ∼76.3 ± 2.1 nM (Figure 1A). The shift shows a sharp concentration dependence similar to cooperative binding, rather than binding of one ADR-2 molecule (Figure 1B). To rule out the possibility that the observed shift in dsRNA was a result of non-specific retardation of dsRNA due to aggregated protein molecules, we made mutations in the KKxxK motif (Figure 1C) at residues that are critical for dsRNA binding in other dsRBDs (45–47). Varying concentrations (up to 1 μM) of the ADR-2 dsRBD mutant did not result in any detectable shift of the dsRNA (Figure 1D), indicating that the observed binding of WT ADR-2 to dsRNA requires a canonical dsRNA-binding motif.

To date, most ADAR proteins have been shown to bind tightly to dsRNA, with apparent affinities in the subnanomolar to 10 nM range (48). To test if the relatively lower affinity of *C. elegans* ADR-2 was a general property of *C. elegans* ADAR proteins or a feature unique to ADR-2, *C. elegans* ADR-1 was expressed in insect cells using BEVS and purified (Supplementary Figure S1B). Binding of recombinant ADR-1 to dsRNA was examined using gel mobility shift assays with the 200 bp dsRNA described above. Incubation of ADR-1 with the dsRNA resulted in a discrete shift and complete binding was observed at a low concentration of ADR-1 (titration up to 8 nM) (Figure 1E). The binding isotherms from three replicate experiments indicate that ADR-1 binds to dsRNA with a *K*_d, app_= 0.43 ± 0.04 nM (Figure 1G and H). This apparent affinity is over 100-fold stronger than ADR-2. One notable exception between ADR-1 and ADR-2 is the presence of two dsRBDs within ADR-1 and only one dsRBD within ADR-2 (Figure 1C and F). To determine if the presence of multiple dsRBDs contributes to the higher affinity of ADR-1 for dsRNA, ADR-1 dsRBD mutants (KKxxK to EAxxA in each dsRBD) were generated, purified and tested by gel mobility shift assay. Consistent with previous *in vivo* binding results (36), an ADR-1 protein with mutations in both dsRBDs showed no detectable binding to dsRNA *in vitro* (Figure 1G and H). Each of the ADR-1 individual dsRBD mutants bound to the dsRNA with slightly reduced affinity compared to WT ADR-1 (Figure 1G and H) (*K*_d, app_= 1.47 ± 0.47 nM for ADR-1 dsRBD1 mutant and 0.95 ± 0.33 nM for ADR-1 dsRBD2 mutant). However, each ADR-1 dsRBD mutant still exhibited ∼50–80-fold stronger affinity toward dsRNA than that of ADR-2, which contains a single dsRBD. Together, these data indicate that the dsRBD of *C. elegans* ADR-2 has a lower affinity for dsRNA in contrast to all previously studied ADARs, including *C. elegans* ADR-1.

### ADR-1 binding to mRNAs is not sufficient to regulate editing *in vivo*

**Figure 2.**
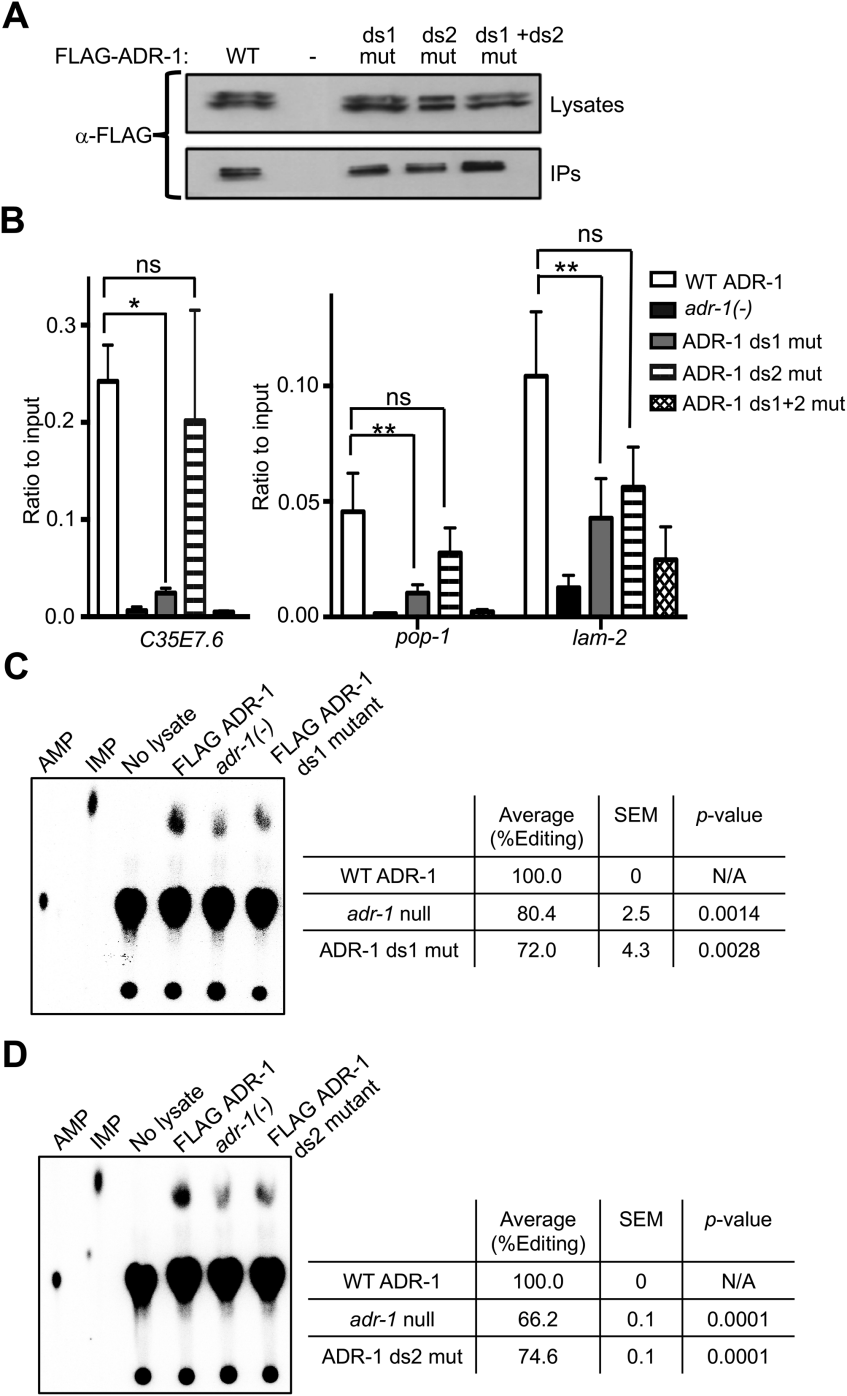
Both dsRBDs of ADR-1 are required to regulate editing *in vivo*. (**A**) Lysates from the indicated worm strains were incubated with α-FLAG magnetic beads. A portion of the lysates before incubation with beads and the IP were subjected to immunoblotting for the FLAG epitope. (**B**) Immunoprecipitates from (A) were treated with proteinase K to release bound RNA. RNA was extracted and reverse transcribed. Bar graph represents the ratio of cDNA in the immunoprecipitates divided by the cDNA in input lysates from the indicated worm strains. Error bars represent SEM for three biological replicates. A one-way ordinary analysis of variance (ANOVA) test was performed. Single and double asterisks indicate significant changes where *P* = 0.05 and *P* ≤ 0.05, respectively, in the RNA binding between WT ADR-1 and ADR-1 dsRBD mutants. (**C** and **D**, left panels) Phosphorimages of TLC plates with ^32^P-AMP and ^32^P-IMP markers and ^32^P-AMP-labeled dsRNA digested to mononucleotides after incubation with the indicated lysates or no lysate, as a negative control. (**C** and **D** right panels) AMP and IMP were quantified using image quant and the % editing was calculated [% Editing = IMPs/ [AMPs + IMPs] and normalized to the amount of editing in the WT FLAG ADR-1 lysate in each replicate. Student’s *t*-test was performed on the average value of three independent replicate experiments for ADR-1 ds1 mutant and two replicate experiments for ADR-1 ds2 mutant. Average % editing, SEM and the *P-*values are shown.

We have previously shown that ADR-1 promotes editing by ADR-2 at specific sites across the transcriptome and this activity requires dsRNA binding by ADR-1 (36). However, considering the higher *in vitro* affinity of ADR-1 for dsRNA (Figure 1) and the fact that ADR-1 is expressed 10-fold higher than ADR-2 at the mRNA level (39), it is unclear how ADR-1 would promote editing *in vivo* and not just compete with ADR-2 for mRNAs. To begin to address this question, we examined ADR-1 binding to dsRNA *in vivo*. We used an established RIP assay where worms are UV-irradiated to crosslink bound RNAs before IP of ADR-1 (36). This RIP assay used transgenic worm lines with WT or mutant ADR-1 with a 3× FLAG epitope at the N-terminus introduced into an *adr-1* null background. These transgenic worm lines express the genomic sequence of ADR-1 under the control of the ADR-1 promoter to mimic endogenous ADR-1 localization and splicing (36). Enrichment of three known edited mRNAs was measured for WT ADR-1 and mutants with KKxxK to EAxxA mutations in each or both dsRBDs and compared to immunoprecipitates from *adr-1(-)* worms. Equal expression and IP of WT ADR-1 and mutants was confirmed by immunoblot (Figure 2A). The remaining immunoprecipitate portion was treated with proteinase K to release bound RNAs that were quantified by reverse transcription quantitative PCR (RT-qPCR). *C35E7.6, pop-1* and *lam-2* were 8–38-fold enriched in the WT ADR-1 immunoprecipitate compared to the immunoprecipitate from the *adr-1(-)* strain (Figure 2B). Furthermore, and consistent with previous data (36), the ADR-1 mutant containing mutations in both dsRBDs did not show a significant enrichment for any of the mRNAs when compared to the immunoprecipitate from the *adr-1(-)* strain. Interestingly, and in contrast to the *in vitro* RNA-binding data, the individual dsRBD mutants of ADR-1 bound differentially to mRNAs *in vivo* (Figure 2B). The ADR-1 dsRBD1 mutant immunoprecipitate had significantly less enrichment for all three mRNAs compared to WT ADR-1, suggesting dsRBD1 is essential for ADR-1 to bind mRNAs *in vivo*. In contrast, the ADR-1 dsRBD2 mutant immunoprecipitate had an enrichment similar to WT ADR-1 for all three mRNAs, suggesting dsRBD2 does not significantly contribute to ADR-1 mRNA binding *in vivo*.

To determine whether ADR-1 binding to mRNA is the only requirement for ADR-1 to promote editing by ADR-2, the individual ADR-1 dsRBD mutant worms were assessed in an established biochemical editing assay (12). For this assay, extracts were prepared from adult worms and incubated with the 200 bp radiolabeled dsRNA corresponding to the *lam-2* 3′ UTR. After incubation of the dsRNA with the worm lysates, the dsRNA was cleaved into single nucleotide monophosphates and subjected to TLC, which separates adenosine (unedited) and inosine (edited) monophosphate. Extracts were prepared from worms lacking *adr-1(-)* and transgenic worms expressing WT ADR-1 and both ADR-1 individual dsRBD mutants. Equal concentrations of the lysates were used in the assay and immunoblot indicated equivalent amounts of the ADR-2-editing enzyme in each lysate (Supplementary Figure S2A and B). Consistent with the ability of ADR-1 to promote editing by ADR-2, extracts from the transgenic FLAG-ADR-1 worms generate significantly more IMP than the extracts from *adr-1(-)* worms (Figure 2C and D). In contrast, the FLAG-ADR-1 dsRBD1 mutant lysate generated a similar amount of IMP as the extract from *adr-1(-)* worms (Figure 2C), indicating that the ability of ADR-1 to bind mRNA *in vivo* is essential for the ability of ADR-1 to promote editing by ADR-2. Surprisingly, the FLAG-ADR-1 dsRBD2 mutant lysate also failed to promote editing by ADR-2 (Figure 2D). As the ADR-1 dsRBD2 mutant was capable of binding mRNA similar to WT (Figure 2B), these data suggest that the dsRBD2 of ADR-1 promotes ADR-2 editing through a function other than RNA binding. Additionally, editing in the ADR-1 dsRBD1+2 mutant lysate is not significantly different from the *adr-1(-)* lysate (Supplementary Figure S2C–E). Together, these data indicate that both dsRBDs of ADR-1 are required to regulate editing *in vivo*.

### ADR-1 forms a complex with ADR-2 independent of dsRNA

**Figure 3.**
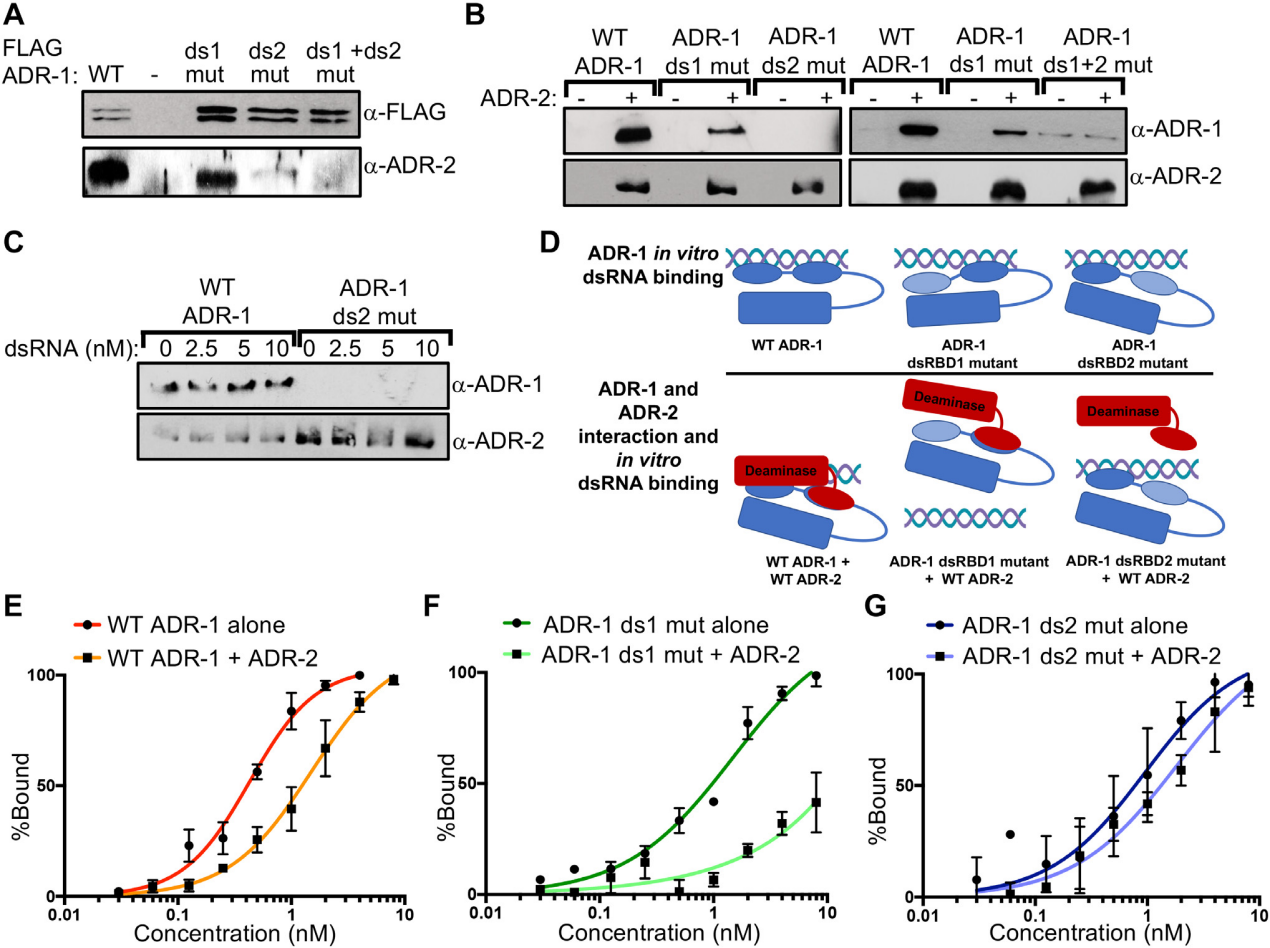
The second dsRBD of ADR-1 is involved in a protein–protein interaction with ADR-2 that allows for binding of the complex to dsRNA. (**A**) Lysates from the indicated worm strains were incubated with α-FLAG magnetic beads. The immunoprecipitates were subjected to SDS-PAGE and immunoblotting with FLAG (ADR-1) and ADR-2 (PA6496) antibodies (*n* = 3 independent biological replicates). (**B**) Magnetic IgG resin was incubated with antibodies specific for ADR-2 (PA6496). After washing, the resin was divided in half and incubated with buffer or WT recombinant ADR-2 protein for an hour. After washing, the resins were incubated with the indicated recombinant ADR-1 proteins, washed and subjected to SDS-PAGE and immunoblotting with ADR-1 and ADR-2 antibodies. Multiple independent replicates were performed for each protein analyzed (*n* = 5 for ADR-1 ds1 and ADR-1 ds2 mutant, *n* = 3 for ADR-1 ds1+2 mutant). (**C**) Similar to the method as described for (B), except for samples were incubated with the indicated concentrations of a 46 bp dsRNA corresponding to sequence from the *lam-2* 3′ UTR when the indicated ADR-1 recombinant proteins were added to the magnetic resin (*n* = 3 independent replicates). The bound proteins were subjected to SDS-PAGE and immunoblotting with ADR-1 and ADR-2 antibodies. (**D**) Model for ADR-1 binding to dsRNA *in vitro* either alone (top panel) or in complex with ADR-2 (bottom panel). (**E** and **F**) Increasing concentrations of ADR-1 and ADR-1+ADR-2 complex were mixed with ^32^P-labeled 200 bp dsRNA corresponding to the sequence from the *lam-2* 3′-UTR (20 pM). Initial and final protein concentrations are marked above gel with adjacent lanes representing a 2-fold difference in protein concentration. The average value for % bound at each concentration from three replicates was used to generate the binding curves for specific binding with Hill slope. (**E**) The binding curve of WT ADR-1 alone (red squares) and ADR-1+ADR-2 complex (orange circles), (**F**) ADR-1 ds1 mutant alone (dark green circles) and ADR-1 ds1 mutant in the presence of ADR-2 (green squares), (**G**) ADR-1 ds2 mutant (dark blue circles) and presence of ADR-2 (blue circles). (**E–G**) Error bars indicate SEM for each point from three independent replicates.

As the second dsRBD of ADR-1 was required to promote editing by ADR-2, but not binding of ADR-1 to mRNA *in vivo*, we sought to determine whether dsRBD2 of ADR-1 might be required for a direct protein–protein interaction with ADR-2. In all kingdoms of life, it has been shown that dsRBPs can interact directly with other dsRBPs via protein–protein interactions between dsRBDs (49,50). In particular, studies on human and fly ADARs indicate that this domain mediates homodimerization between ADARs and heterodimerization of ADARs with other dsRBPs (30,31,51). As a first step to determine if dsRBD2 of ADR-1 mediates a protein–protein interaction with ADR-2, an *in vivo* co-IP assay was performed. Briefly, ADR-1 was immunoprecipitated using the FLAG epitope present at the N-terminus of WT or mutant ADR-1. An interaction between ADR-1 and ADR-2 was examined by immunoblot of the ADR-1 immunoprecipitates with an ADR-2 antibody. ADR-2 was detected in the immunoprecipitate from worms expressing FLAG-ADR-1, but not in the immunoprecipitate from *adr-1(-)* worms, indicating an *in vivo* interaction (Figure 3A). However, it is possible that this interaction is indirect and mediated by RNA binding. To examine this possibility, the interaction between ADR-2 and the ADR-1 mRNA-binding mutant, ADR-1 dsRBD1 EAxxA, was examined. Interestingly, the ADR-1 dsRBD1 mutant was capable of pulling down ADR-2, suggesting that dsRNA binding by ADR-1 is not required for the *in vivo* interaction of ADR-1 and ADR-2 (Figure 3A). In contrast, IP of the ADR-1 dsRBD2 mutant and the ADR-1 dsRBD1 + dsRBD2 mutant resulted in almost no detectable ADR-2 protein in the immunoprecipitate (Figure 3A). Importantly, immunoblotting for ADR-2 in these lysates indicated that the absence of ADR-2 in the ADR-1 dsRBD2 mutant and ADR-1 dsRBD1 + dsRBD2 mutant immunoprecipitates is not caused by a decrease in ADR-2 protein expression in the mutant lysates compared to the WT ADR-1 lysate (Supplementary Figure S3). As the ADR-1 dsRBD2 mutant binds mRNA similar to WT ADR-1 but is unable to promote RNA editing by ADR-2, these data suggest that the second dsRBD of ADR-1 may be important for mediating a protein–protein interaction with ADR-2.

To determine if ADR-1 directly interacts with ADR-2, an *in vitro* pulldown assay was performed using purified proteins and a reverse immunoprecipitation order. In this assay, recombinant ADR-2 was immobilized on magnetic beads using an ADR-2 antibody and then either recombinant WT ADR-1 or ADR-1 dsRBD mutants were incubated with the ADR-2-bound resin. After stringent washing, the presence of ADR-1 and ADR-2 on the resin was determined by immunoblotting. Using this assay, recombinant WT ADR-1 was pulled down by recombinant ADR-2, but not resin alone (Figure 3B). Consistent with the *in vivo* co-IP, the ADR-1 dsRBD1 mutant was pulled down by recombinant ADR-2 (Figure 3B). In contrast, the ADR-1 dsRBD2 mutant and the dsRBD1 + dsRBD2 mutants did not interact with ADR-2 in the pulldown assay (Figure 3B). These data are consistent with the *in vivo* co-IP (Figure 3A) and suggest the second dsRBD of ADR-1 serves as the major protein–protein interaction site for ADR-1 and ADR-2.

It is important to note that in both the *in vivo* and *in vitro* co-IP assays, the ADR-1 dsRBD1 mutant appears to have a reduction in binding to ADR-2, suggesting that this domain may partially also contribute to the interaction between ADR-1 and ADR-2. As dsRBD1 of ADR-1 is the major contributor of mRNA binding by ADR-1, it is possible that RNA binding contributes to the observed interaction between ADR-1 and ADR-2. As one test of this possibility, the WT recombinant proteins were treated with micrococcal nuclease before performing the *in vitro* co-IP assay. Importantly, the ADR-1 and ADR-2 interaction detected in the *in vitro* co-IP assay was not altered by treatment of the recombinant proteins with micrococcal nuclease (Supplementary Figure S4), suggesting that RNA is not critical for mediating the interaction between ADR-1 and ADR-2. To further probe if dsRNA binding by ADR-1 could contribute to the observed interaction between ADR-1 and ADR-2, the *in vitro* co-IP assay was performed in the presence of dsRNA with the ADR-1 dsRBD2 mutant, which lacks the ability to physically interact with ADR-2 but has similar dsRNA binding to WT ADR-1 (Supplementary Figure S5). The presence of increasing concentrations of dsRNA did not affect the interaction of the ADR-1 dsRBD2 mutant and ADR-2 (Figure 3C), indicating that ADR-1 binding to dsRNA is not sufficient to mediate the interaction between ADR-1 and ADR-2. Interestingly, the presence of dsRNA (over a range of concentrations, including saturation of both molecules) did not inhibit the interaction of WT ADR-1 and ADR-2, suggesting that the ADR-1 and ADR-2 interaction occurs independent of RNA binding.

Together, our *in vitro* and *in vivo* data suggest that ADR-1 and ADR-2 directly interact through ADR-1 dsRBD2, and ADR-1 dsRBD1 is critical for dsRNA binding *in vivo*. As a further test of this model (Figure 3D), gel mobility shifts were performed with WT and mutant ADR-1/ADR-2 complexes and the *lam-2* 200 bp dsRNA, as described above (Figure 1). Co-incubation of WT ADR-1 and ADR-2 with the dsRNA resulted in a discrete shift (Supplementary Figure S6A). Analysis of the binding isotherms from three replicate experiments indicates that an ADR-1/ADR-2 complex binds to dsRNA with an approximate *K*_d, app_= 1.50 nM (Figure 3E), which is very similar to the affinity of the individual dsRBD mutants of ADR-1 (Figure 1 and Supplementary Figure S6B). This shift in affinity of ADR-1 in the presence of ADR-2 is consistent with a model that ADR-2 binding to dsRBD2 of ADR-1 forces ADR-1 to bind dsRNA with dsRBD1 only. To test this hypothesis, the gel shift assays were performed with the ADR-1 dsRBD mutants in complex with WT ADR-2. As expected, the presence of ADR-2 did not allow the ADR-1 dsRBD1 + dsRBD2 mutant to bind dsRNA (data not shown). ADR-2 significantly reduced dsRNA binding by ADR-1 dsRBD1 mutant, consistent with this dsRBD being the primary dsRNA interaction (Figure 3F, *P*-value = 0.05, unpaired *t*-test). In contrast, the addition of ADR-2 did not have any significant change in binding of the ADR-1 dsRBD2 mutant (Figure 3G). Together these data suggest that ADR-1 interacts with ADR-2 via the second dsRBD of ADR-1 and the complex binds dsRNA via the first dsRBD of ADR-1.

### ADR-1 is required for ADR-2 to bind most mRNAs *in vivo*

**Figure 4.**
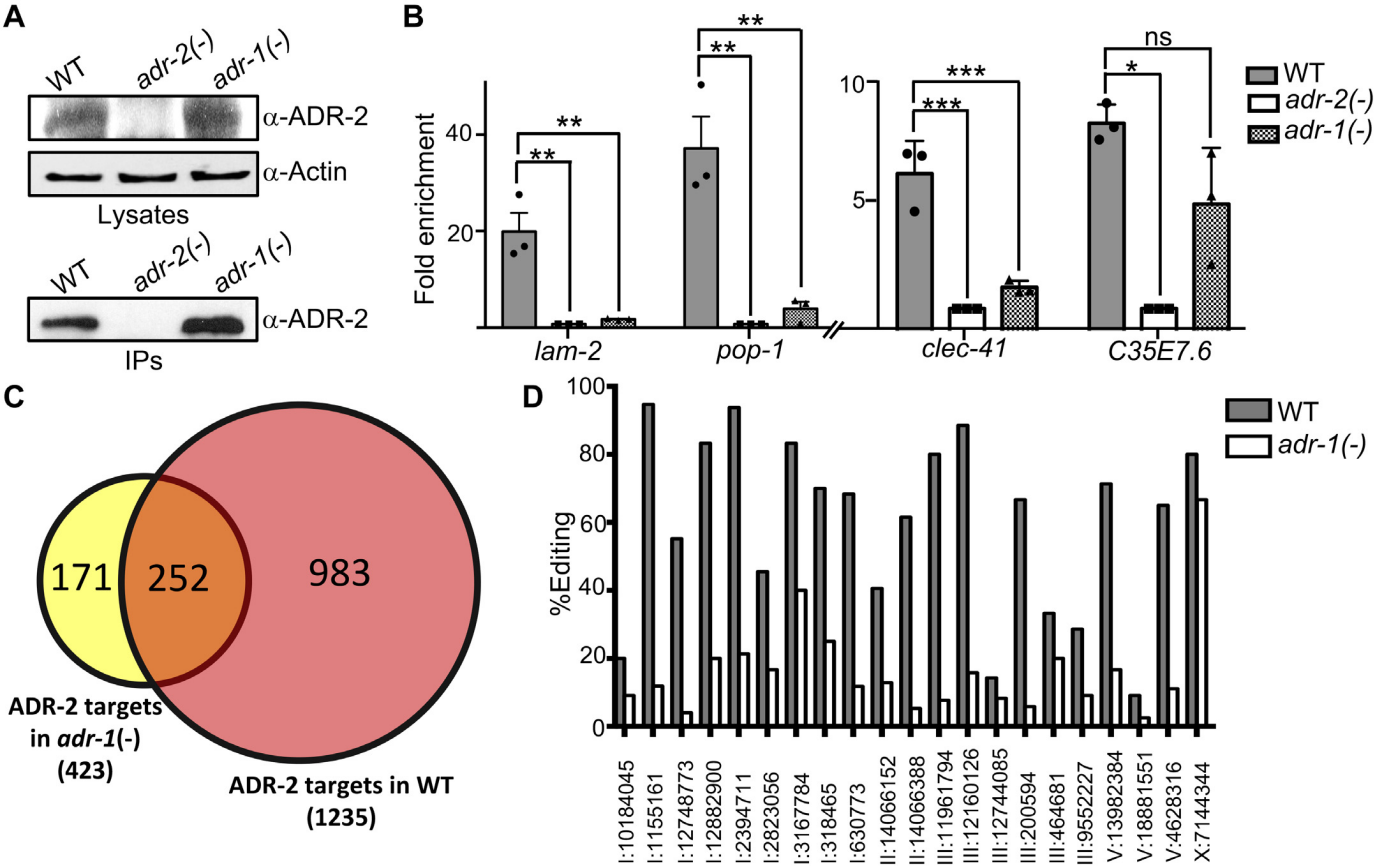
ADR-1 is required for ADR-2 to stably bind to a majority of mRNAs *in vivo*. (**A**) Lysates from the indicated worm strains were incubated with magnetic beads, which were pre-incubated with ADR-2 antibodies. A portion of the lysates before incubation with beads and the immunoprecipitation (IP) were subjected to immunoblotting for ADR-2. Actin was used as a loading control for lysates. (**B**) Immunoprecipitates from (A) were treated with proteinase K to release bound RNA. RNA was extracted and reverse transcribed. Bar graph represents the fold enrichment of cDNA present in the immunoprecipitates divided by the cDNA present in input lysates from the indicated worm strains and normalized to the same ratio in *adr-2(-)* worms. Error bars represent SEM for three biological replicates. A one-way ordinary ANOVA test was performed. Single, double and triple asterisks indicate significant changes where *P* < 0.05, *P* < 0.005 and *P* ≤ 0.001, respectively. (**C**) Circle diagrams representing the 1235 ADR-2-bound targets identified from ADR-2 RIP-seq in WT worms (red) and the 423 ADR-2-bound targets identified from ADR-2 RIP-seq in *adr-1(-)* worms (yellow), with targets identified in both datasets represented by orange overlapping region. (**D**) Editing levels from the RNA-seq data for representative sites (see Supplementary Table S4 for all sites) in 21 transcripts that are bound to ADR-2 in WT worms, but not *adr-1(-)* worms.

While the *in vitro* dsRNA-binding experiments suggest ADR-1 is important for the ability of ADR-2 to bind dsRNA, we sought to determine whether this same requirement could be observed *in vivo*. To examine binding of ADR-2 to mRNA *in vivo*, we used an RIP assay for ADR-2 that was recently developed by our laboratory (37). To directly examine whether the presence of ADR-1 influenced ADR-2 binding to mRNA, immunoprecipitates from UV-irradiated WT and *adr-1(-)* lysates were compared. Importantly, ADR-2 was efficiently immunoprecipitated from both WT and *adr-1(-)* lysates, but not from lysates of *adr-2(-)* worms (Figure 4A). RT-qPCR showed a 22-fold enrichment of *lam-2* mRNA in the WT immunoprecipitates compared to the *adr-2(-)* immunoprecipitates, indicating that ADR-2 stably associates with the *lam-2* mRNA in WT worms (Figure 4B). However, using *adr-1(-)* worm lysates, ADR-2 failed to enrich *lam-2*, suggesting that, *in vivo*, ADR-2 is unable to interact with the *lam-2* mRNA in the absence of ADR-1 (Figure 4B). To test the influence of ADR-1 on ADR-2 binding to other known targets, we examined *pop-1* and *clec-41*, two mRNAs in which ADR-1 binding promotes RNA editing (36,37). *Pop-1* and *clec-41* mRNA were enriched in the ADR-2 immunoprecipitates from WT worms compared to those from *adr-2(-)* worms by 37- and 7-fold, respectively, but both showed dramatically lower enrichment from *adr-1(-)* worm lysates (Figure 4B). In contrast to the ADR-1 dependence of the ADR-2 interaction with these three mRNAs, the ADR-2 immunoprecipitates from both WT and *adr-1(-)* lysates exhibited similar enrichment for the *C35E7.6* mRNA, suggesting that stable association of ADR-2 with *C35E7.6* mRNA does not require ADR-1 (Figure 4B). Interestingly, the *C35E7.6* mRNA was previously shown to be bound by ADR-1 *in vivo*, and binding of ADR-1 to *C35E7.6* mRNA increased in the absence of *adr-2* (36), suggesting that ADR-1 and ADR-2 may compete for binding to some mRNAs.

Examination of four known edited mRNAs using the ADR-2 RIP assay suggests that ADR-1 promotes stable binding of ADR-2 to a subset of mRNAs. To determine whether this mechanism to promote ADR-2 mRNA binding is widespread in the transcriptome, the ADR-2 immunoprecipitates from WT and *adr-1(-)* lysates, as well as the negative control immunoprecipitates from *adr-2(-)* lysates, were subjected to high-throughput RNA sequencing (RIP-seq). In addition, high-throughput sequencing was performed on RNA isolated from each worm strain before immunoprecipitation, which serves as a normalization for the transcript expression in the worm strains. To identify RNAs that immunoprecipitated with ADR-2, differential enrichment of transcripts in the WT immunoprecipitates (normalized to the transcripts present in the WT worms) compared to the immunoprecipitates from *adr-2(-)* worms (normalized to the transcripts present in the *adr-2(-)* worms) was performed for two biological replicates using DESeq2 (52). From this analysis, 1235 transcripts were significantly enriched in the ADR-2 immunoprecipitates from WT worms (Figure 4C and Supplementary Table S1). Importantly, 62% of these transcripts have previously been reported to contain editing sites, which is more than expected by random chance (42%), suggesting these are in fact *bona fide* ADR-2 target RNAs (Supplementary Table S1). To determine whether ADR-1 was required for ADR-2 association with target RNAs, ADR-2 RIP-seq from *adr-1(-)* worms was analyzed and identified 423 transcripts significantly enriched in the ADR-2 immunoprecipitates (Figure 4C and Supplementary Table S2). When comparing these 423 transcripts to the 1235 ADR-2-associated RNAs identified from WT worms, a majority (252) were common. The lack of enrichment for the other 983 transcripts bound in WT worms was not due to a lack of expression or differential expression of the transcripts between WT and *adr-1(-)* worms (Supplementary Table S3). These data indicate that ADR-1 is required for 80% of the transcripts to associate with ADR-2 in WT worms. Interestingly, 171 transcripts were enriched in the ADR-2 immunoprecipitates from *adr-1(-)*, but not WT worms, suggesting that in the absence of *adr-1*, ADR-2 may associate with some unique target RNAs (Figure 4C).

To determine whether the ability of ADR-1 to direct ADR-2 to specific transcripts correlates with functional consequences on RNA, we performed *de novo* editing site identification and quantification using our recently developed software, *SAILOR* (37) on the RNA-sequencing (RNA-seq) datasets from the lysates before ADR-2 immunoprecipitation described above. Although these datasets are not sequenced to the depth needed to identify all edited sites, from the WT RNA-seq, we identified 578 editing sites within the 1406 ADR-2-bound transcripts (both circles in Figure 4C, data not shown). To determine the impact of ADR-1 on editing, we quantified editing at these sites in the input RNA-seq datasets from the ADR-2 RIP experiment in *adr-1(-)* worms. Within 23 ADR-2-bound transcripts, 260 editing sites had significant read coverage in the *adr-1(-)* RNA-seq to quantify editing using *SAILOR* (Supplementary Table S4). Of these 23 transcripts, 21 transcripts contain 189 sites that have decreased editing (>5%) in *adr-1(-)* worms (Figure 4D and Supplementary Table S4). Importantly, all 21 of these transcripts exhibit ADR-1-dependent ADR-2 binding. Together, these data indicate that ADR-1 directs the substrate specificity of ADR-2 *in vivo* and increasing the association of ADR-2 with target RNAs is the mechanism by which ADR-1 promotes editing.

## DISCUSSION

In this study, we provide evidence that the *C. elegans* ADAR enzyme, ADR-2, functions as a complex with the deaminase-deficient ADAR family member, ADR-1. Using both *in vitro* dsRNA binding and transcriptome-wide examination of ADR-2 association with cellular RNAs, we found that ADR-1 has >100-fold greater affinity for dsRNA compared to ADR-2, and stable interaction of ADR-2 with most target mRNAs *in vivo* requires ADR-1. Using WT and mutant ADR-1 proteins expressed in the worm and as recombinant proteins, we were able to demonstrate that ADR-1 primarily uses the first dsRBD to bind mRNA *in vivo*, despite being able to use each individual dsRBD to bind to dsRNA *in vitro* (Figures 1 and 2). These data suggest that studying ADAR binding to dsRNA *in vitro* is unlikely to reveal specificity determinants, which is consistent with recent data on another dsRBP, ILF3 (53). In addition, these data suggested that the second dsRBD of ADR-1 might be performing a different role *in vivo*. Our co-IP analysis from worms revealed that mutation of the second dsRBD of ADR-1 abolished the interaction with ADR-2 without significantly affecting the ability of ADR-1 to interact with mRNA (Figures 2 and 3). Together, these data indicate that ADR-1 and ADR-2 physically interact independent of RNA binding.

We propose a model in which ADR-1 and ADR-2 form a heterodimer primarily via the second dsRBD of ADR-1 and the dsRBD of ADR-2 (Graphical abstract). This heterodimeric complex binds cellular RNAs via the first dsRBD of ADR-1, which promotes stable association of ADR-2 with substrates to allow for efficient editing. Our proposed molecular mechanism, in which a co-factor not only recognizes, but also tethers an ADAR enzyme to its substrate, has not been demonstrated in any other organism. Human and *Drosophila* ADAR-editing enzymes have been reported to form dimers (30,51,54,55). However, whether dimerization is required for editing activity (56) and/or mediated by interactions with RNA (46) for ADAR function *in vivo* is both controversial and unknown (57). A particular complication in previous studies of dimerization mediated by dsRBDs is that the KKxxK motif in the dsRBD is classically defined as important for dsRNA binding and when mutated to EAxxA resulted in both disruption of the protein–protein interaction and altered *in vitro* dsRNA binding. However, our *in vivo* results clearly demonstrate that mutation of the KKxxK motif in the second dsRBD of ADR-1 does not significantly impact ADR-1 binding to mRNA but does disrupt the protein–protein interaction with ADR-2 (Figures 2 and 3). To the best of our knowledge, this is the first time that ADAR dsRBD mutants have been studied for effects on *in vivo* binding to specific mRNAs. It will be interesting to expand this analysis transcriptome-wide to determine if the second dsRBD of ADR-1 contributes to recognition of specific RNAs or if this domain is only used for protein–protein interactions.

The use of a co-factor to promote RNA binding of a modification enzyme to substrates is widespread outside of the ADAR family. For example, the human and fly RNA-binding proteins, RBM15 and RBM15B, bind the RNA methyltransferase machinery and recruit it to transcripts for methylation of specific adenosines (58,59). In addition, cytidine to uridine editing of mRNA by the apolipoprotein B mRNA editing catalytic polypeptide (APOBEC1) family requires an RNA-binding co-factor, RBM47 (60). Our data indicate that, similar to the function of mammalian RBM47, *C. elegans* ADR-1 both physically interacts with ADR-2 and promotes stable binding of the complex to RNA to allow ADR-2 to properly edit. However, previous studies have shown that ADR-1 is not required for ADR-2 to edit all adenosines (12,36,61) and our RIP studies identified 423 transcripts that associate with ADR-2 in the absence of ADR-1 (Supplementary Table S3). These data suggest that the mode of substrate recognition by ADR-2 differs among various transcripts. However, the low apparent affinity of ADR-2 for dsRNA raises the question of whether or not ADR-2 is capable of using its dsRBD to bind targets *in vivo*. The amino acid sequence alignment of dsRBDs from ADAR family members indicates that the ADR-2 dsRBD has a valine within the α1 helix instead of a glutamate residue, which is known to be one of the two dsRNA minor groove interaction sites for dsRBDs (62). Mutation of this glutamate residue to alanine in the dsRBP, Staufen, abolished dsRNA binding (45). Future studies to examine ADR-2 association with cellular RNAs in different tissues and at different points in development will be critical to understanding the ability of ADR-2 to directly bind transcripts and/or associate with RNA-binding co-factors other than ADR-1. In our current *in vitro* studies, the binding of ADR-2 alone to dsRNA appeared to exhibit cooperative binding (sharp transition from unbound to fully bound dsRNA, Hill coefficient of ∼4.4), suggesting the weak affinity of ADR-2 might only be physiological relevant in cells with high expression of ADR-2. In contrast, the higher affinity to dsRNA for ADR-2 in complex with ADR-1 exhibits less cooperative binding to dsRNA (Hill coefficient of ∼1.2), suggesting that the interaction of ADR-2 with ADR-1 in order to bind substrates may be most important in cells with low expression of ADR-2. Further *in vitro* studies of the dynamics of ADR-2 interaction with different dsRNAs may provide insight into how ADR-2 binds and edits certain transcripts in the absence of ADR-1.

Many types of DNA and RNA modifications are catalyzed by a complex of two proteins, where one subunit catalyzes the modification reaction and the partner subunit is not enzymatically active (63–65). Our study is the first to demonstrate such a complex for the ADAR family of enzymes. However, the related adenosine deaminases that act on tRNA (ADATs) function as a heterodimer, where ADAT2 is the catalytic subunit and ADAT3 increases ADAT2 binding to tRNA and may contribute to the zinc coordination required for adenosine deamination (66). While our regulatory mechanism for the ability of ADR-1 to promote editing by ADR-2 involves a complex similar to ADAT2/3, our data also suggest that ADR-2 has functions that do not require, and may even compete with, ADR-1 mRNA binding. Therefore, unlike ADAT2, which requires ADAT3 for A-to-I editing of tRNA, *C. elegans* ADR-2 is also able to function as an A-to-I editing enzyme independent of complex formation with ADR-1. Future work aimed at dissecting the cellular pathways that control dynamics of complex formation between ADR-1 and ADR-2 will be critical for understanding the different modes of substrate recognition by ADAR enzymes.

In summary, our results indicate that both ADR-1 binding to mRNA and physical interactions between ADR-1 and ADR-2 underlie the regulatory mechanism by which ADR-1 promotes ADR-2 editing of specific transcripts. The conservation of editing-deficient ADAR proteins opens the possibility that human ADAR-editing enzymes could also interact with deaminase-deficient ADAR family members, or other dsRBPs, to recognize specific target mRNAs *in vivo*. In this regard, it was recently reported that binding of human ADAR2 to a specific transcript (CTN-RNA) is enhanced by ADAR1 binding to the same transcript, and vice versa (67,68). The authors propose that ADAR1 and ADAR2 interact in an RNA-dependent manner as a reduction in both proteins is observed in the co-IP treated with RNase (67). An alternative possibility is that the two proteins physically associate to bind some transcripts and co-localize to other transcripts (in an RNA-dependent manner), wherein treatment with nuclease would show a reduction in the ability of the two proteins to co-immunoprecipitate. In this regard, our previous co-IP study of *C. elegans* ADR-1 and ADR-2 showed a reduction in protein levels when the immunoprecipitates were treated with RNase, but with a RNase-stable portion of both proteins in the immunoprecipitate (36). However, in this study, we were able to identify a mutant of ADR-1 that had similar mRNA binding as WT ADR-1 but exhibited a defect in binding to ADR-2, indicating the two proteins interact in an RNA-independent manner. Future studies of mammalian ADARs should examine how effects of RNA-binding mutants of one ADAR family member impact the ability to co-immunoprecipitate other ADAR family members and how these RNA-binding mutants impact RNA binding to test whether physical association between mammalian ADARs contributes to recognition of specific substrates.

## DATA AVAILABILITY

Processed and raw data for all high-throughput sequencing experiments described here are available from the Gene Expression Omnibus (accession GSE112367).

## Supplementary Material

Supplementary Data
