# Supplementary material for: A protein–protein interaction underlies the molecular basis for substrate recognition by an adenosine-to-inosine RNA-editing enzyme
Source: Nucleic Acids Res. 2018 Sep 7;46(18):9647–59. doi: 10.1093/nar/gky800 (PMC6182170; doi:10.1093/nar/gky800)
Supplement: Supplementary Data [file gky800_supplemental_files.zip › Rajendren, Hundley, Supplementary information, NAR resubmission, clean.pdf]

Supplementary methods:

MNase treatment of proteins and Immunoprecipitation:

The purified recombinant proteins are mixed with MNase reaction buffer (50mM Tris-Cl, 5mM $CaCl_2$, pH 7.9), and the mixture was treated with MNase (NEB) at a concentration of 20U/μl for 30 minutes at 37°C. Following MNase treatment, co-IPs were performed as described (see materials and methods) and analyzed by SDS-PAGE and western blotting.

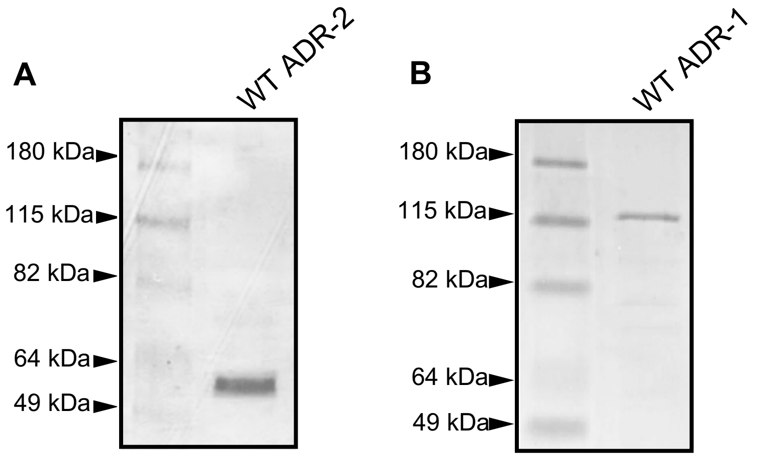

Figure S1: Purification of *C. elegans* ADARs using a baculovirus expression system. (A) The coding region of *adr-2* was cloned into the pKL MC2 vector immediately downstream of a 10-histidine tagged maltose-binding protein (MBP) and expressed in Hi5 cells. Lysates were incubated with Amylose resin, then ADR-2 was cleaved from the MBP tag with 3C protease, the cleaved protein was incubated with Heparin Sepharose to further purify the protein (See Materials and methods). The final protein preparation was analyzed by 10% SDS-PAGE and stained with Coomassie. The predicted size of the protein after the cleavage of MBP tag is 55 kDa. **(B)** The coding region of *adr-1* was cloned into the pFastBacHTB vector downstream of a 6-histidine tag and expressed in Sf9 cells. Lysates were incubated with Ni-NTA resin to purify the protein (See Materials and methods). The final protein preparation was analyzed by 10% SDS-PAGE and stained with Coomassie. The predicted size of the protein, including the polyhistidine tag is 110 kDa.

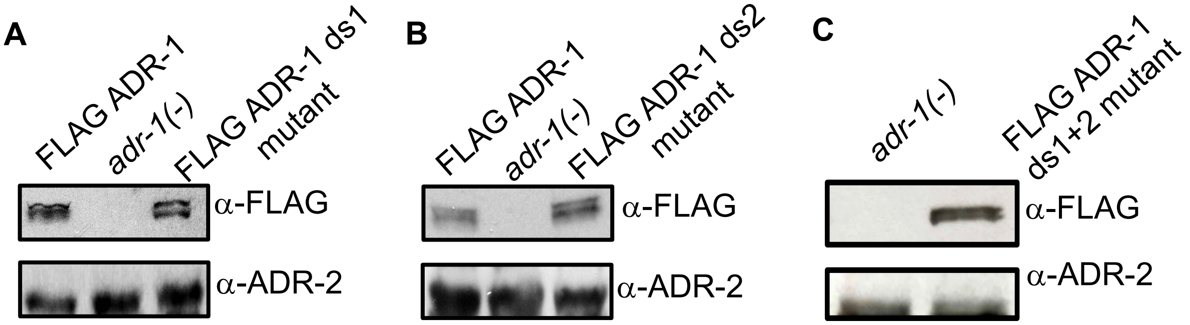

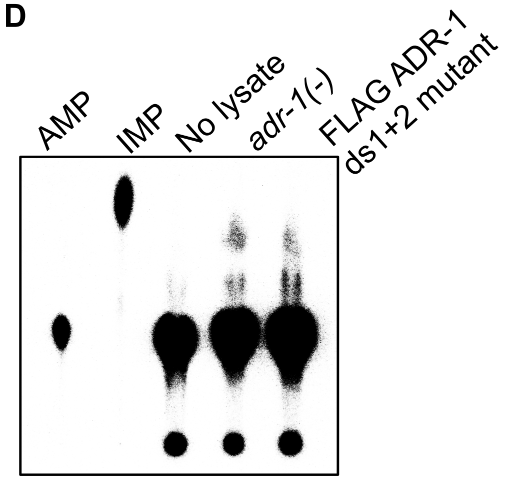

E

	**Average**	**SEM**	***p*-value**
adr-1(-)	1.00	0.00	N/A
ADR-1 ds1+2 mut	0.96	0.04	0.32

Figure S2: *C. elegans* ADAR protein expression in lysates for deamination assays. (A, B, C) For the *in vitro* deamination assay, worm extracts were made from the transgenic worms expressing FLAG-ADR-1, FLAG ADR-1 ds1 mutant, FLAG ADR-1 ds2 mutant and FLAG ADR-1 ds1+2 mutant proteins along with *adr-1*(-) worms. Equal concentrations of the deamination lysates of the indicated strains were analyzed by SDS-PAGE and immunoblotting against FLAG (ADR-1) and ADR-2 epitopes. **(D)** Phosphorimage of thin layer chromatography (TLC) plates with ^{32}P-AMP and ^{32}P-IMP markers and ^{32}P-AMP-labelled dsRNA digested to mononucleotides after incubation with the indicated lysates or no lysate, as a negative control. **(E)** AMP and IMP were quantified using image quant and the % editing was calculated [% Editing = IMPs/ [AMPs + IMPs] and normalized to the *adr-1*(-) lysate in each replicate. Student's t-test was performed on the average value of three independent replicate experiments.

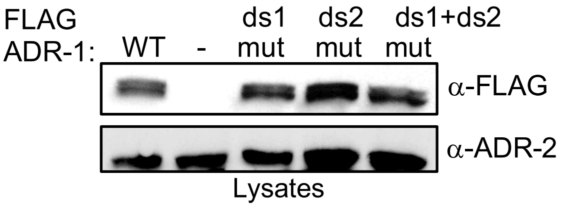

Figure S3: Expression of *C. elegans* ADAR proteins in lysates for co-immunoprecipitation assays. Lysates from the indicated worm strains were incubated with α-FLAG magnetic beads. A portion of the lysates before incubation with beads were subjected to immunoblotting with the FLAG and ADR-2 (IU529) antibodies.

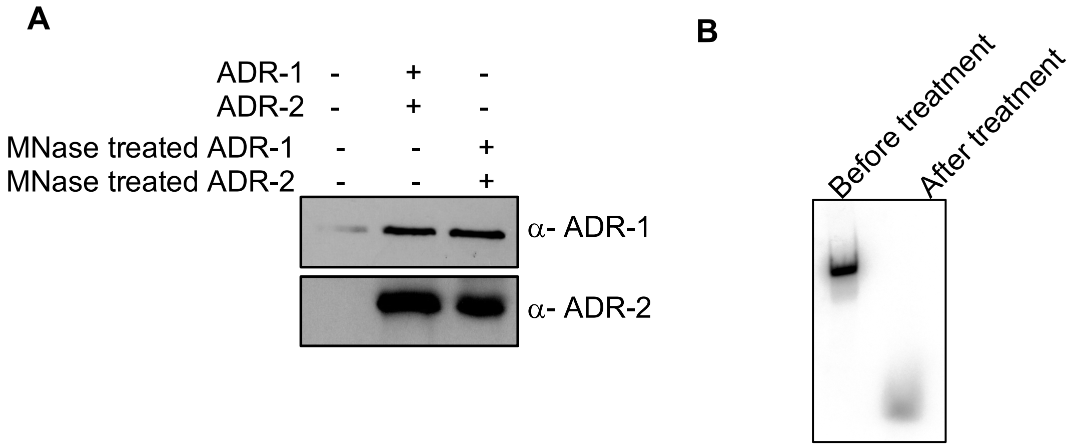

Figure S4: Nuclease treatment does not abolish the interaction of ADR-1 and ADR-2. (A) Magnetic IgG resin was incubated with antibodies specific for ADR-2 (PA6496). After washing unbound antibodies, the resin was divided into three equal portions and incubated with buffer or wildtype (WT) recombinant ADR-2 protein (6 nM) that had been treated (+) or not (-) with Micrococcal Nuclease (MNase) at a concentration of 20U/µl. After washing, the resins were incubated with WT recombinant ADR-1 protein (3 nM) that had been treated (+) or not (-) with MNase, washed and subjected to SDS-PAGE and immunoblotting with ADR-1 and ADR-2 antibodies. (N=2) **(B)** 10nM Radiolabeled dsRNA (200 bp *lam-2* 3'UTR) was mixed with MNase reaction buffer and the mixture was treated with MNase (NEB) at a concentration of 20U/µl for 30 minutes at 37°C. Following MNase treatment, 50% of the reaction was loaded into a Native 6% (19:1 acrylamide/ bisacrylamide (Biorad) gel. Electrophoresis was performed at 4°C at 200 V for 25 minutes with 1X TBE running buffer. Gels were dried, exposed to phosphorimager screens overnight and autoradiographed.

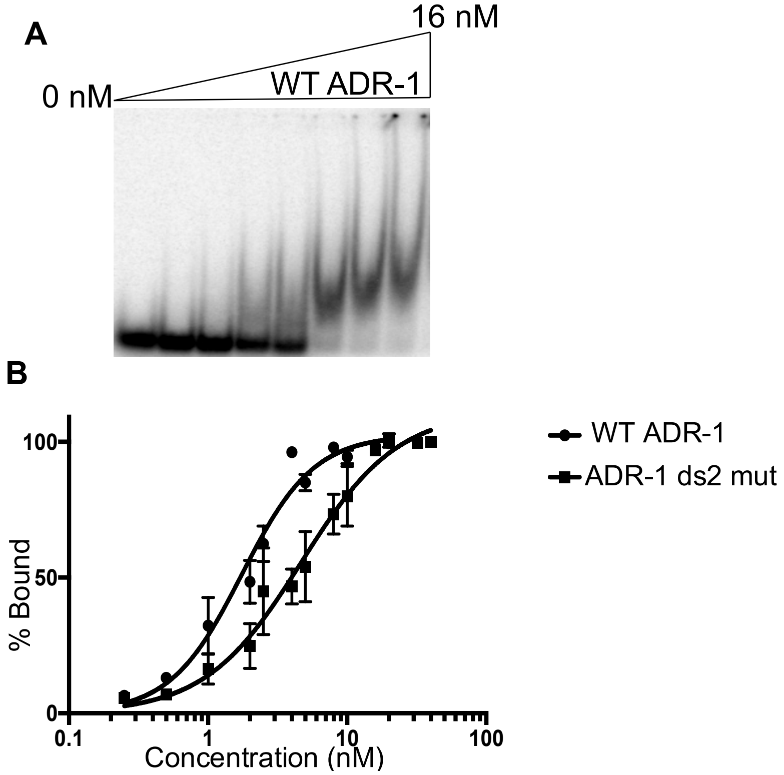

	$K_{d,app}$ (nM)
WT ADR-1	1.75 ± 0.15
ADR-1 ds2 mut	4.67 ± 0.70

Figure S5: Wildtype and dsRBD2 mutant ADR-1 binding to a 46 bp dsRNA *in vitro*. **(A)** Increasing concentrations of wildtype (WT) ADR-1 were incubated with ^{32}P-labeled 46 bp dsRNA corresponding to the sequence from the *lam-2* 3'-UTR (20 pM). Initial and final protein concentrations are marked above gel with adjacent lanes representing a two-fold difference in protein concentration. **(B)** RNA binding isotherms for gel shift assays conducted with the 46 bp dsRNA and the indicated ADR-1 proteins. The average value for % bound at each protein concentration was used to generate the binding curve. Error bars indicate standard error mean (SEM) for each point from three replicates of each assay. Values for the dissociation constant ($K_{d,app}$) were calculated from the RNA binding isotherms.

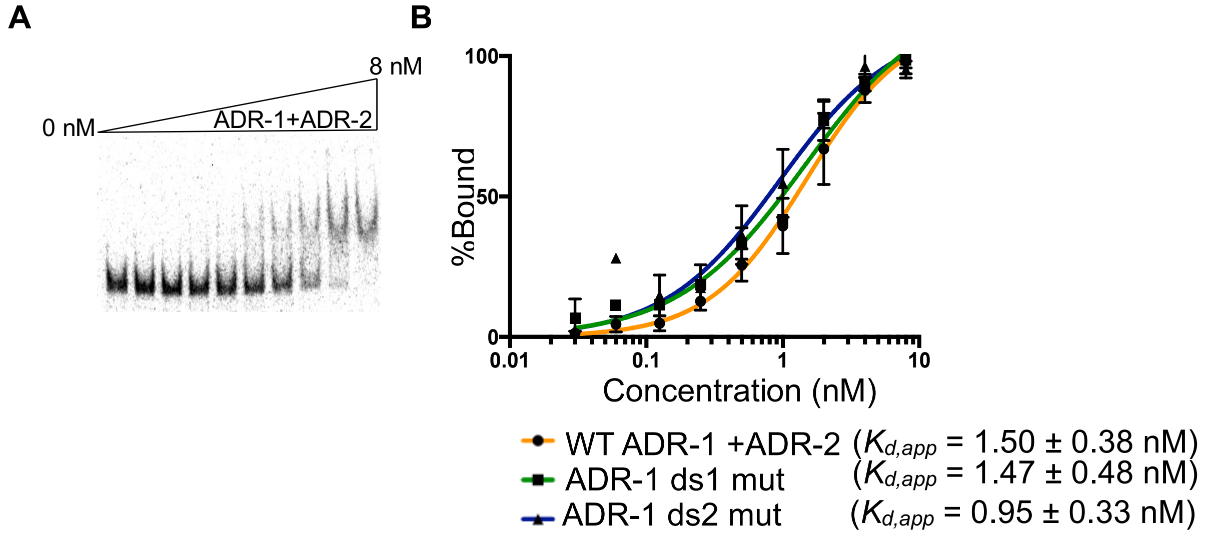

Figure S6: ADR-1/ADR-2 complex binds to dsRNA *in vitro*. (A) Increasing amounts of purified recombinant wildtype (WT) ADR-1 and ADR-2 proteins were mixed and incubated on ice. After 20 minutes, ^{32}P-labeled 200 bp dsRNA from *lam-2* 3'UTR (20 pM) was added to each tube and incubated on ice for another 30 minutes. Initial and final protein concentrations are marked above gel with adjacent lanes representing a two-fold difference in protein concentration. **(B)** RNA binding isotherms for gel shift assays conducted with the 200 bp RNA and the WT ADR-1/ADR-2 complex or the indicated ADR-1 proteins. The average value for % bound at each protein concentration was used to generate the binding curve. Error bars indicate standard error mean (SEM) for each point from three replicates of each assay. Values for the dissociation constant ($K_{d,app}$) were calculated from the RNA binding isotherms.

Table Legends:

Table S1: ADR-2 targets identified from ADR-2 RIP-Seq in wildtype worms, related to Figure 4C. Genes that exhibit a differential enrichment (p-value < 0.05 and a fold enrichment ($\log_2$fold)> 0.5) between the ADR-2 IPs from wildtype (WT) and *adr-2(-)* worms are listed by wormbase gene name (Gene) and common genetic annotation (Name). DESeq2 values for ((IP WT/Input WT)/(IP *adr-2(-)*/Input *adr-2(-)*) are listed (log2 Fold Change) as well as both the raw (pvalue) and corrected (padj) *p-values* for each gene. Read counts from DESeq2 for IPs and inputs of two biological replicates for each strain are listed in columns F-M. Each transcript was checked against a list of previously published edited targets in *C. elegans*[1] (column N). The ADR-2 RIP-Seq data from *adr-1(-)* worms (Table S2) was used to determine whether these targets are dependent on ADR-1 to bind ADR-2 *in vivo* (column O).

Table S2: ADR-2 targets identified from ADR-2 RIP-Seq in *adr-1(-)* worms, related to Figure 4C. Genes that exhibit a differential enrichment (p-value < 0.05 and a fold enrichment ($\log_2$fold)> 0.5) between the ADR-2 IPs from *adr-1(-)* worms and *adr-2(-)* worms are listed by wormbase gene name (Gene) and common genetic annotation (Name). DESeq2 values for ((IP *adr-1(-)* /Input *adr-1(-)*)/(IP *adr-2(-)*/Input *adr-2(-)*) are listed (log2 Fold Change) as well as both the raw (pvalue) and corrected (padj) *p-values* for each gene. Read counts from DESeq2 for IPs and inputs of two biological replicates for each strain are listed in columns F-M. Each transcript was checked against a list of previously published edited targets in *C. elegans*[1] (column N). The ADR-2 RIP-Seq data from wildtype (WT) worms (Table S1) was used to determine whether these targets are bound to ADR-2 in WT worms (column O).

Table S3: Gene expression in *adr-1(-)* worms compared to wildtype worms. Expression of all genes in the input RNA-sequencing datasets from the two biological replicates of wildtype (WT) and *adr-1(-)* worms are listed by wormbase gene name (Gene) and common genetic annotation (Name). DESeq2 values for (Input WT/Input *adr-1(-)*) are listed (log2 Fold Change) as well as both the raw (pvalue) and corrected

(padj) *p-values* for each gene. Read counts from DESeq2 for inputs of two biological replicates for each strain are listed in columns F-I.

Table S4: Computationally identified editing sites in both WT and *adr-1(-)*, related to Figure 4D. The input RNA-seq datasets from the ADR-2 RIP assays conducted in WT and *adr-1*(-) worms were analyzed by *SAILOR*, a previously published software program that predicts A-to-I editing sites[1]. High confidence editing sites (>0.99 confidence, $\geq$ 5 reads per site) identified in WT dataset are listed by chromosome and nucleotide position (Column A). The percent editing for each site was calculated by the number of reads containing G divided by the total number of reads at a given site for both the wildtype (Column B) and *adr-1(-)* (Column C) datasets. The difference in editing levels between WT and *adr-1(-)* and the annotated region where the editing site falls within the transcript are listed in Columns D and E, respectively. Editing sites are assigned (see Materials and Methods) to both a wormbase gene name (wbgene) and common genetic annotation (gene). The lists of target genes from the ADR-2 RIP-Seq in *adr-1(-)* worms (Table S3) and wildtype worms (Table S1) were used to identify whether the editing sites are located in transcripts bound by ADR-2 (Columns H and I, respectively). Editing sites that have reduced levels of editing (> 5%) in the absence of *adr-1* are highlighted in green.

Table S5: Sequences of all primers and synthetic oligonucleotides, related to Experimental Procedures. Primers used in this study for PCR amplification and qRT-PCR analysis.

References:

1 Deffit, S. N. *et al.* The C. elegans neural editome reveals an ADAR target mRNA required for proper chemotaxis. *Elife* **6**, doi:10.7554/eLife.28625 (2017).
